# Theophylline-7β-d-Ribofuranoside (Theonosine), a New Theophylline Metabolite Generated in Human and Animal Lung Tissue

**DOI:** 10.3390/pharmaceutics9030028

**Published:** 2017-08-14

**Authors:** Daniel S. Sitar, James M. Bowen, Juan He, Angelo Tesoro, Michael Spino

**Affiliations:** 1 Departments of Pharmacology and Therapeutics, Internal Medicine, and Pediatrics and Child Health, Max Rady College of Medicine, Centre on Aging, and College of Pharmacy, University of Manitoba, Winnipeg, MB R3E 0T6, Canada; 2 Leslie Dan Faculty of Pharmacy, University of Toronto, Toronto, ON M5S 3M2, Canada; bowenj@mcmaster.ca (J.M.B.); info@biopharmaservices.com; (J.H.); amtesoro@rogers.com (A.T.); mspino@apopharma.com (M.S.)

**Keywords:** theophylline, theophylline metabolism, lung, microsomes, theophylline-7β-d-ribofuranoside, theonosine

## Abstract

While assessing the ability of mammalian lung tissue to metabolize theophylline, a new metabolite was isolated and characterized. The metabolite was produced by the microsomal fraction of lungs from several species, including rat, rabbit, dog, pig, sheep and human tissue. Metabolite production was blocked by boiling the microsomal tissue. This new metabolite, theophylline-7β-d-ribofuranoside (theonosine), was confirmed by several spectral methods and by comparison to an authentic synthetic compound. Tissue studies from rats, rabbits, dogs, and humans for cofactor involvement demonstrated an absolute requirement for NADP and enhanced metabolite production in the presence of magnesium ion. It remains to be demonstrated whether theonosine may contribute to the known pharmacological effects of theophylline.

## 1. Introduction

The lung is known to play an important role in the metabolism of several drugs [1,2,3]. Most enzymes so far identified are found in lower concentrations in the lung than in the liver, but the lung receives all of the cardiac output in comparison to the liver, which receives about one quarter of it. Some drug metabolizing enzymes found in the lung are not found in the liver, suggesting the potential for lung tissue to produce unique drug metabolites [4].

More than 40 cell types have been identified in lung tissue, and drug metabolism has been detected in both endothelial and epithelial cells. Pulmonary endothelium, comprising about 70 m^2^, is in direct contact with blood, and is active in regulating biogenic amines, prostaglandins, vasoconstrictive peptides, lipids, and nucleotides [5]. Removal of vasoconstrictive amines by the lung is more effective than by the liver [6]. Alveolar macrophages and epithelial tissue are active in drug biotransformation, particularly Clara, type I and type II cells [7]. Although it is clear that the lung can metabolize endogenous substances and drugs, its contribution to the overall metabolism of drugs remains to be better characterized [8,9,10].

**Figure 1:**
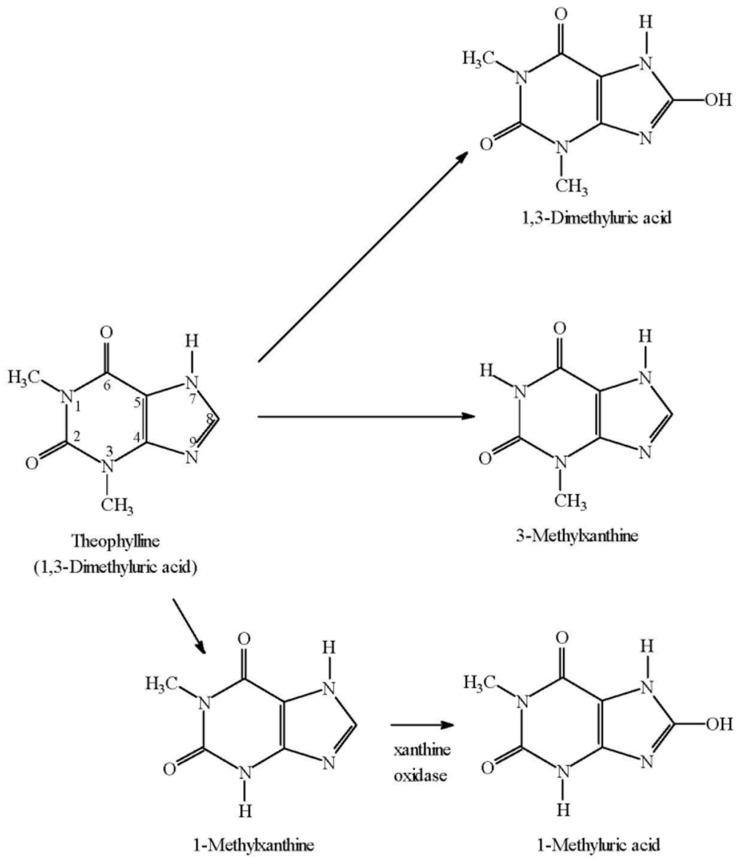
Summary of theophylline metabolism from urinary excretion data.

Theophylline has been used therapeutically as a bronchodilator for more than 80 years [11]. Its metabolic disposition in humans was first reported by Brodie et al. [12]. Following a therapeutic dose, only 85% has been accounted for by measurement of known metabolites and unchanged drug excreted in urine [13] (Figure 1).

It has been observed previously that theophylline clearance is increased in patients with cystic fibrosis [14]. While testing the hypothesis that lung tissue from cystic fibrosis patients contributed to its more rapid kinetic disposition, we determined that mammalian lung microsomes from five species, including humans, biotransformed theophylline to the newly characterized metabolite, theophylline-7β-d-ribofuranoside (theonosine). This paper describes the isolation, identification, and characterization of production of this new conjugated theophylline metabolite.

## 2. Experimental Section

### 2.1. Tissue Sources and Collection

Experiments with animal lungs were completed with salvage tissue from animals killed for studies completed in accordance with protocols approved by the University of Toronto Animal Care Committee. Human lung tissue was obtained from surgical waste resulting from partial pulmonary lobectomy for carcinoma or from lung transplant recipients. Normal tissue was excised. Following resection, tissue was washed as described below for lungs from animal species and flash frozen in liquid nitrogen. Approval for use of human tissue was obtained from the Pathology Department, The Toronto Hospital (Toronto General Division), and the Review Board of the Hospital for Sick Children. Permission to conduct the study was obtained from the Human Subjects Review Committee, Hospital for Sick Children. Lungs and other tissues were removed from mongrel dogs, mixed-breed pigs, New Zealand white rabbits, and mixed-breed sheep under approved operating room conditions immediately following the death of the animals. Tissue was gently flushed with 1.15% *w*/*v* KCl solution to remove blood, and tissue was flash frozen in liquid nitrogen or placed on ice and transported immediately to the laboratory for preparation of microsomes.

### 2.2. Methods

#### 2.2.1. Preparation of Microsomes

Frozen tissue was thawed and homogenized within one week of acquisition. Microsomes were prepared as described previously [15]. Briefly, tissue was weighed and placed in an ice-cold solution of 1.15% *w*/*v* KCl and 0.1 M sodium phosphate buffer pH 7.4. Excessive connective tissue was removed, and the remaining tissue was minced with scissors and resuspended in a fresh volume of KCL/phosphate buffer (1:4 *w*/*v*). The suspension was homogenized with a Brinkman Polytron^®^ instrument using a PT10ST probe and centrifuged at 10,000× *g* at 4 °C for 20 min to remove cellular debris, nuclei, and mitochondria. The supernatant fluid was decanted into ultracentrifuge tubes and centrifuged at 105,000× *g* at 4 °C for 60 min (L8-80 Beckman Ultracentrifuge) to isolate the microsomal fraction. The resulting supernatant was removed and the microsomal pellet was resuspended in fresh KCl/phosphate buffer with a Teflon-glass Potter–Elvehjem homogenizer. This suspension was centrifuged again at 105,000× *g* at 4 °C for 60 min to isolate the microsomal fraction. The supernatant was removed, and the microsomal pellet was resuspended in fresh 0.2 M potassium phosphate buffer (pH 7.4) at a ratio of 1 g lung/mL of buffer solution. Microsomal protein was determined by the method of Lowry et al. [16].

#### 2.2.2. In Vitro Metabolism Studies

Metabolic incubation studies for microsomal metabolism of theophylline were completed as follows. The incubation mixture, 0.50 mL, contained 40 mM pH 7.4 potassium phosphate buffer, 0.23% *w*/*v* KCl, 2 mM MgCl_2_, 0.4 mM NADP, 4 mM glucose-6-phosphate, 0.4 U/mL glucose-6-phosphate dehydrogenase (G-6-PD), approximately 500 μg of lung microsomal protein, and varying concentrations of theophylline. All components, excepting the substrate, were preincubated for 5 min at 37 °C before the addition of theophylline substrate. The reaction was incubated in a shaking water bath at 120 rpm (Model G 86, New Brunswick Scientific Co. Inc., Edison, NJ, USA) for various time periods up to 60 min in initial studies, and for 30 min once kinetic characteristics of metabolite production were established. The reaction was stopped by the addition of 3.0 mL of acetone containing 20 μL of glacial acetic acid and β-hydroxyethyltheophylline as the internal standard. The treated incubation mixture was centrifuged at 1000× *g* for 10 min and the resulting supernatant removed and evaporated under a stream of nitrogen. The residue was reconstituted with 0.2 mL of distilled water and an aliquot injected for determination of theophylline metabolism by high performance liquid chromatography (HPLC) using a stepwise linear gradient elution [17]. Separation of theophylline, theonosine, and the internal standard β-hydroxyethyltheophylline was excellent with retention times of 17.0, 19.5 and 22.5 min, respectively. The limit of quantitation for theonosine was 2 ng, corresponding to a range of 0.05–1.0 pmol/mg protein/min enzyme activity. Peak height was linear to 700 pmol theonosine/reaction tube. The new metabolite was collected from the eluting solvent of the HPLC instrument and was further purified by adsorption to reversed-phase Bond Elut^®^ columns (Varian Canada Inc., Mississauga, ON, Canada). Columns were washed with water to remove phosphate buffer. The purified metabolite was then eluted from the column by desorption with acetonitrile. The resulting compound was then subject to analyses by ultraviolet, Fourier-transformed infrared, proton nuclear magnetic resonance, and mass spectral instrumentation.

In order to determine the conditions contributing to production of the new theophylline metabolite, incubations were conducted as follows: (i) in room air; (ii) under carbon monoxide and nitrogen atmospheres to assess requirements for cytochromes P450 and oxygen; (iii) after preincubation of microsomes at 37 °C for 45 min to inactivate flavine monooxygenase [18]; (iv) with boiled microsomes to determine if the reaction was enzyme catalyzed; and (v) in the presence and absence of various cofactors described above in the standard incubation mixture. Inhibition studies were completed in the presence of varying concentrations of cimetidine, caffeine, methimazole, SKF525A, indomethacin, and antipyrine. To evaluate substrate specificity for the conjugation reaction, theonosine production was determined in the presence of added ribose, adenine, adenosine, inosine, and guanosine.

#### 2.2.3. Synthesis of Theophylline-7β-d-Ribofuranoside

This compound was synthesized, purified, and chemically and spectrally characterized as described previously [19].

#### 2.2.4. Chemicals

Theophylline, glucose-6-phosphate, glucose-6-phosphate dehydrogenase, β-hydroxyethyltheophylline, caffeine, D-ribose, adenine, adenosine, inosine, guanosine, indomethacin, antipyrine, and methimazole were obtained from Sigma Chemical Co. (St. Louis, MO, USA). Cimetidine and SKF525A were gifts from Smith, Kline and French Laboratories (Toronto, ON, Canada). Other chemicals were analytical grade, excepting acetonitrile, which was HPLC grade.

#### 2.2.5. Data Analyses

Kinetic analyses of our metabolic data were conducted using the classical assumptions of Michaelis and Menten. Data were transformed by the Lineweaver–Burk, Eadie–Hofstee, and Cornish–Bowden procedures to determine apparent *K*_m_, *V*_max_, *K*_i_, and potential mechanisms to explain the inhibitions observed. Data are presented as mean ± SD. Statistical analyses were done with appropriate parametric tests, and a two-tailed *p*-value < 0.05 was the level at which a difference was accepted.

## 3. Results and Discussion

### 3.1. Microsomal Preparation

Lung microsomes prepared by the method described above resulted in the following yield of microsomal protein (mg/g lung tissue) in each of the following species: rat: 7.9 ± 2.1 (*n* = 7); rabbit: 6.0 ± 1.9 (*n* = 13); pig: 5.8 ± 0.7 (*n* = 3); sheep: 6.6 (*n* = 1); dog: 4.9 ± 1.8 (*n* = 4); human: 2.6 ± 1.3 (*n* = 21). Lung microsomal protein concentration for patients with cystic fibrosis (3.1 ± 1.3 mg/g, *n* = 8) and patients without cystic fibrosis (2.3 ± 1.2 mg/g, *n* = 13) were not different (*p* < 0.21).

### 3.2. Identification and Characterization of the Production of the New Theophylline Metabolite

**Figure 2:**
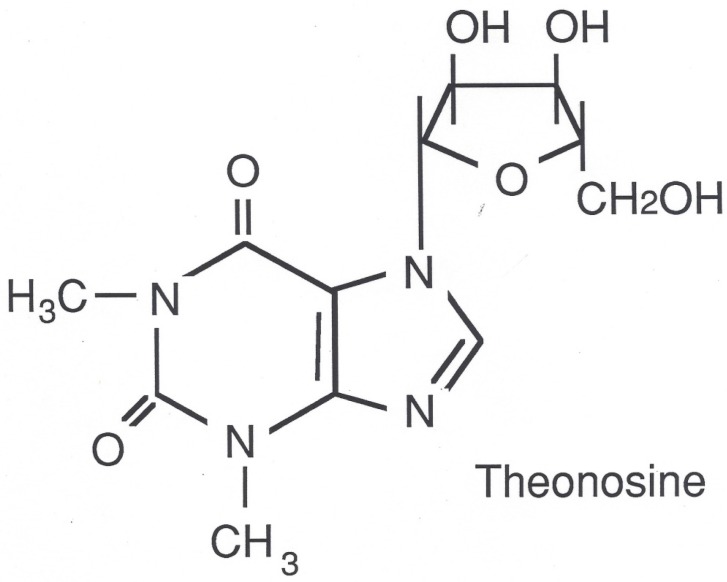
Theonosine structure.

The lung microsomal fraction among species differed both qualitatively and quantitatively in the production of theophylline metabolites. However, all species tested generated the ribosylated conjugate metabolite theonosine (Figure 2).

There are few published data with respect to theophylline metabolism by lung tissue. Kröll et al. evaluated ^14^C-theophylline disposition in a perfused guinea pig lung preparation, but were unable to detect any metabolites in the circulation. Uptake of the radiolabel by their lung preparation was insufficient to allow evaluation of metabolite production [20].

The wavelength maximum for theonosine’s ultraviolet absorbance was 274 nm, very similar to that for theophylline, 272 nm, and much lower than that produced by oxidation of theophylline to 1,3-dimethyluric acid, 287 nm. This spectral finding confirms the initial report by Myake et al. [19]. Its elution from the HPLC column after theophylline confirms the findings of IJzerman et al. [21].

Incubation studies of theophylline with rabbit lung microsomes suggested that metabolite production was enzymatic, since it did not occur with boiled microsomes. No enzyme activity was detected in the post-microsomal supernatant, and the 10,000× *g* pellet produced only about 5% of the metabolite compared to lung microsomes. There was an absolute requirement for NADP, but NADP, NADPH, or an NADPH generating system were effective in supporting production of this newly isolated theophylline metabolite from lung microsomes. It was noteworthy that the reaction occurred with or without G-6-PD as long as NADP or NADPH was present (data not shown).

Studies to determine optimal conditions for production of theonosine with dog lung microsomes demonstrated that maximal activity occurred at 55 °C, with product formation being three times greater than that observed at 37 °C. The addition of Mg^+2^ up to a concentration of 6 mM increased production of the metabolite by almost two-fold, after which production plateaued.

The generation of theonosine was not inhibited by carbon monoxide, SKF525A, or antipyrine, indicating that it was not mediated by cytochromes P450. Preincubation of microsomes at 37 °C for 45 min, or coincubation with methimazole, did not affect production of the new metabolite, indicating a lack of contribution by amine oxidase. Incubation with indomethacin did not inhibit metabolite production, suggesting that cyclo-oxygenase was not involved (data not shown).

**Figure 3:**
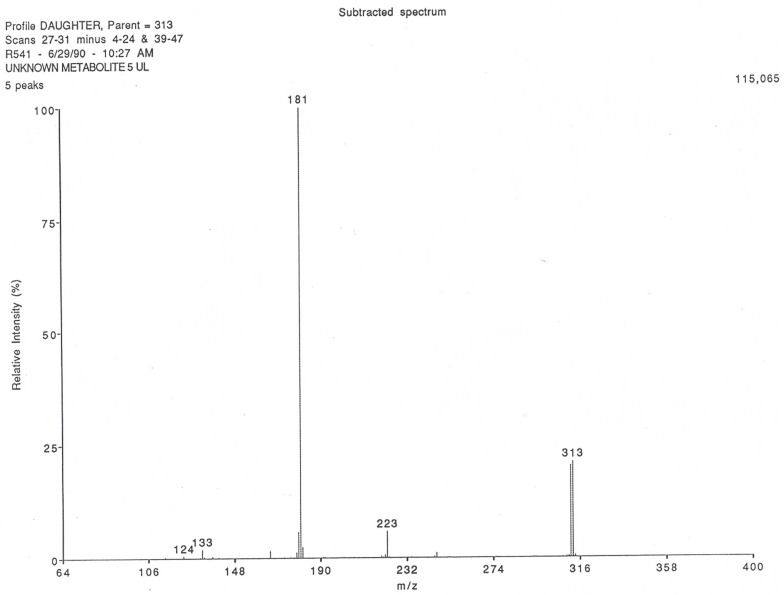
HPLC/MS/MS chemical ionization spectrum of theonosine.

High performance liquid chromatography/mass spectral/mass spectral analysis of the metabolite gave an M + 1 ion of 313 (23% abundance) (Figure 3) with a base peak at mass 181, corresponding to that for pure theophylline when it was analyzed by the same method.

Direct probe insertion of theonosine into the source gave an electron impact mass spectrum with a molecular ion at m/e 312 (3.4% abundance) and a base peak at m/e 180, corresponding to the molecular ion fragment of theophylline obtained by the identical technique. Fourier-transformed infrared analysis indicated the presence of a sugar conjugate. Subsequently, proton nuclear magnetic resonance analysis of a solution in deuterated water at 400 MHz suggested that the metabolite contained a 5-carbon sugar attached to nitrogen with spectral characteristics consistent with ribose.

From these data, we tentatively identified the metabolite as theophylline-7-riboside and propose the name theonosine to reflect its similarity to adenosine. Chemical synthesis and spectral analysis confirmed the identity of the metabolite as theophylline-7β-d-ribofuranoside (theonosine) [19].

Our data reveal for the first time that metabolic transformation of theophylline to a newly identified conjugate metabolite, theonosine occurs in lung tissue from several mammalian species.

Theonosine has been detected following lung microsomal incubation from all species studied to date, including human (Table 1).

Metabolite production is linear to 1000 μg of microsomal protein per incubation tube and for 60 min. In human lung microsomes, apparent theophylline *K*_m_ for theonosine production was determined as 51 ± 24 μM with an apparent *V*_max_ of 63 ± 37 pmol/mg protein/min.

**Figure 4:**
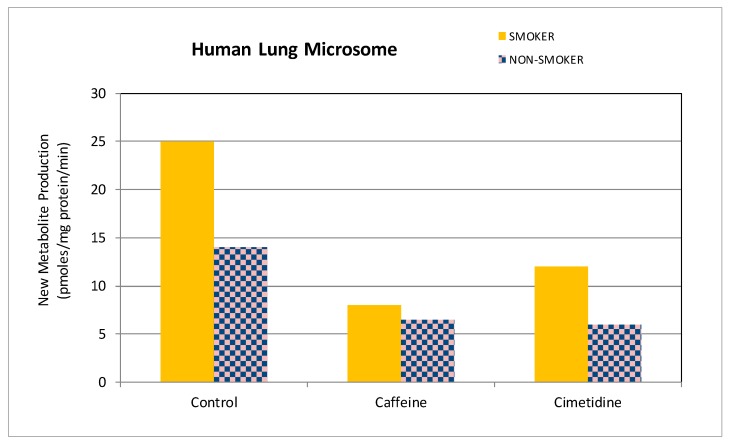
Inhibition of theonosine production by caffeine and cimetidine in human lung microsomes.

When incubated with dog, rabbit, pig, or human lung microsomes, 2 mM cimetidine inhibited theonosine production by about 50% with 1 mM theophylline as the substrate concentration. Studies with caffeine indicated a similar degree of inhibition of theonosine production for an equivalent molar ratio of inhibitor to substrate in the incubation medium. Inhibitory effect data from studies with representative human lung tissue are presented in Figure 4. These data suggest the potential involvement of CYP1A1/2 in the ribosylation of theophylline, since theonosine production appears to be greater in microsomal tissue from smokers [8,9,10]. However, lack of inhibition of theonosine production by carbon monoxide does not favour such an explanation.

**Figure 5:**
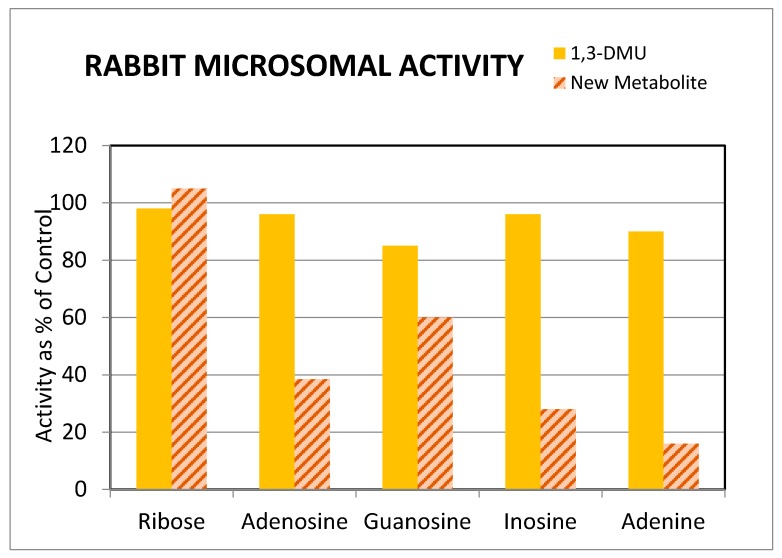
Inhibition of theonosine production by purinergic nucleosides in rabbit lung microsomes.

Finally, we studied the effects of 0.5 mM adenine and three other nucleosides on the production of theonosine by rabbit lung microsomes. These purinergic compounds all inhibited theonosine production in the following order of decreasing potency: adenine, inosine, adenosine, and guanosine. A representative graph of these inhibitions relative to the production of 1,3-dimethyluric acid in the incubation mixture is presented in Figure 5. Studies with adenine and caffeine on the mechanism of inhibition indicated that it was not competitive, but a mixed inhibition as reflected by Dixon and Cornish–Bowden plots (data not shown). Further studies are required to more completely define the mechanisms by which inhibition of theonosine production is occurring.

While conjugation of drugs with ribose in mammals is not common, it has been described for allopurinol [22,23,24,25]. Elion and Hitchings presented data suggesting that the liver and kidney may be the sites of production of allopurinol and oxypurinol ribosides in the rat [22]. The enzyme responsible for ribose conjugation of these drug substances has been identified as purine nucleoside phosphorylase, and its presence has been demonstrated in guinea pig small intestine and in human erythrocytes [23]. In preliminary studies in rat, dog, and rabbit tissue, in vitro, our laboratory has also demonstrated theonosine production from tissues other than the lung (unpublished observations). Further studies are required to conclusively demonstrate that this same enzyme is responsible for the conversion of theophylline to theonosine.

### 3.3. Potential for Pharmacological Activity of Theonosine

There is preliminary in vitro evidence that xanthine-7-ribosides bind to A_1_-type adenosine receptors [26]. These conjugates have been identified as adenosine antagonists by this research group, although theophylline-7β-d-ribofuranoside is considerably less potent than theophylline in its interaction at the A_1_ adenosine receptor. Subsequent to this initial report, evidence has been presented that theophylline-7-ribosides may in fact be partial agonists at the A_1_ adenosine receptor [21,27]. These observations raise the possibility that theonosine may contribute to the bronchodilator effect of theophylline, since its production occurs within the lung.

## 4. Conclusions

Our studies have identified a new metabolite for theophylline, theophylline-7β-d-ribofuranoside (theonosine). These studies also demonstrate that theophylline serves as an additional substrate for the uncommon metabolic pathway of conjugation with ribose in mammalian species, including humans. The biological significance of this metabolic reaction warrants exploration.

## Figures and Tables

**Table 1:** Production of theophylline-7β-d-ribofuranoside (theonosine) by lung microsomal tissue from various mammalian species incubated with 1 mM theophylline. Data are presented as mean ± SD when *n* > 2.

Species	Theonosine Production (pmol/mg protein/min)
Dog (*n* = 5)	31 ± 15
Sheep (*n* = 1)	32
Rabbit (*n* = 8)	11 ± 5
Rat (*n* = 7)	41 ± 10
Pig (*n* = 3)	40 ± 19
Human (*n* = 16)	18 ± 11
